# Methods and design of a 10-week multi-component family meals intervention: a two group quasi-experimental effectiveness trial

**DOI:** 10.1186/s12889-016-3908-x

**Published:** 2017-01-09

**Authors:** Catherine Rogers, Sarah E. Anderson, Jamie S. Dollahite, Tisa F. Hill, Chris Holloman, Carla K. Miller, Keeley J. Pratt, Carolyn Gunther

**Affiliations:** 1 Department of Human Sciences, Human Nutrition Program, The Ohio State University, 325 Campbell Hall, 1787 Neil Avenue, Columbus, OH 43210 USA; 2 Division of Epidemiology, The Ohio State University, 336 Cunz Hall, 1841 Neil Avenue, Columbus, OH 43210 USA; 3 Division of Nutritional Sciences, Cornell University, 408 Savage Hall, Ithaca, NY 14853 USA; 4 Division of Nutritional Sciences, Cornell University, 342 MVR, Ithaca, NY 14853 USA; 5 Department of Statistics, The Ohio State University, 404 Cockins Hall, 1958 Neil Avenue, Columbus, OH 43210 USA; 6 Department of Human Sciences, Human Nutrition Program, The Ohio State University, 347B Campbell Hall, 1787 Neil Avenue, Columbus, OH 43210 USA; 7 Department of Human Sciences, Human Development and Family Science Program, The Ohio State University, 130B Campbell Hall, 1787 Neil Avenue, Columbus, OH 43210 USA; 8 Department of Human Sciences, Human Nutrition Program, The Ohio State University, 313 Campbell Hall, 1787 Neil Avenue, Columbus, OH 43210 USA

**Keywords:** Family meals, Child diet, Weight status, Behavioral intervention, Childhood obesity prevention

## Abstract

**Background:**

Given the ongoing childhood obesity public health crisis and potential protective effect of family meals, there is need for additional family meals research, specifically experimental studies with expanded health outcomes that focus on the at-risk populations in highest need of intervention. Future research, specifically intervention work, would also benefit from an expansion of the target age range to include younger children, who are laying the foundation of their eating patterns and capable of participating in family meal preparations. The purpose of this paper is to address this research gap by presenting the objectives and research methods of a 10-week multi-component family meals intervention study aimed at eliciting positive changes in child diet and weight status.

**Methods:**

This will be a group quasi-experimental trial with staggered cohort design. Data will be collected via direct measure and questionnaires at baseline, intervention completion (or waiting period for controls), and 10-weeks post-intervention. Setting will be faith-based community center. Participants will be 60 underserved families with at least 1, 4–10 year old child will be recruited and enrolled in the intervention (*n* = 30) or waitlist control group (*n* = 30). The intervention (Simple Suppers) is a 10-week family meals program designed for underserved families from racial/ethnic diverse backgrounds. The 10, 90-min program lessons will be delivered weekly over the dinner hour. Session components include: a) interactive group discussion of strategies to overcome family meal barriers, plus weekly goal setting for caregivers; b) engagement in age-appropriate food preparation activities for children; and c) group family meal for caregivers and children. Main outcome measures are change in: child diet quality; child standardized body mass index; and frequency of family meals. Regression models will be used to compare response variables results of intervention to control group, controlling for confounders. Analyses will account for clustering by family and cohort. Significance will be set at *p* < 0.05.

**Discussion:**

This is the first experimentally designed family meals intervention that targets underserved families with elementary school age children and includes an examination of health outcomes beyond weight status. Results will provide researchers and practitioners with insight on evidence-based programming to aid in childhood obesity prevention.

**Trial registration:**

NCT02923050. Registered 03 October 2016. Retrospectively registered.

## Background

The American Academy of Pediatrics recommends participation in family meals as a childhood obesity prevention strategy due to the literature demonstrating a protective effect of participation in healthy mealtime routines on child diet and weight [1]. However, the current evidence linking family meals with improved child dietary intake (increased fruit and vegetable intake, decreased sugar-sweetened beverage (SSB) intake) and weight status (decreased body mass index (BMI; (weight (kg)/height (m)^2^)) z-score) has significant limitations. The majority of the family meals literature – specifically in the area of childhood obesity prevention – represents observational studies, demonstrating only an associative relationship of family meals with child diet and weight status [2–5]. What’s more, racial and ethnic differences have been highly understudied; given that the segment of the United States (US) child population with high prevalence of obesity is racial and ethnic minorities [6], it has been suggested that this is an area in which additional research is needed. Similarly, the existing family meals intervention research (i.e., studies designed specifically to examine the cause and effect relationship between family meals and child diet and weight status), while strong with regard to study design, is limited and primarily targets non-Hispanic White children (8 to 12 years old), particularly from well-educated families [7, 8]. In addition, the majority of the current research fails to examine the child health impact of family meals beyond BMI (e.g., central adiposity and blood pressure (BP)), with only a small number of studies including additional outcomes (e.g., disordered eating) [9–12]. Given the ongoing childhood obesity public health crisis [13] and the potential protective effect of family meals, there is need for additional family meals research, specifically experimental studies with expanded health outcomes that focus on the at-risk populations in highest need of intervention. Future research, specifically intervention work, would also benefit from an expansion of the target age range to include younger children (4–7 year olds), who are laying the foundation of their eating patterns [14], and are capable of participating in family meal preparations [15].

The purpose of this paper is to address this gap in the literature by presenting the objectives and research methods of a 10-week multi-component family meals intervention study, Simple Suppers, aimed at eliciting positive changes in child dietary intake and weight status. The Simple Suppers study is a two group quasi-experimental trial with staggered cohort design that targets underserved families with elementary school age children (4–10 years) and includes an examination of health outcomes beyond weight status.

## Methods

### Objectives and hypotheses

The objectives of this study with related hypotheses will be as follows:

1. Assess the impact of Simple Suppers on children and caregivers of participating families relative to children and caregivers of families in the control group.
  Hypothesis 1.1. Diet quality, BMI z-scores and BMI, waist circumference (WC) z-scores and WC, and BP z-scores and BP will improve more from baseline to post-intervention among children and caregivers, respectively, participating in the intervention than in the controls.
  Hypothesis 1.2. Diet quality, BMI z-scores and BMI, WC z-scores and WC, and BP z-scores and BP improvements will be maintained during the follow-up period among children and caregivers, respectively, participating in the intervention.

Objective 2. Assess the impact of Simple Suppers on the family meals environment of participating families relative to the controls.
  Hypothesis 2.1. Frequency of family meals (breakfast and dinner), TV viewing during meals, and eating family meals in a dining area will improve more from baseline to post-intervention among families participating in the intervention than in the controls.
  Hypothesis 2.2. Frequency of family meals (breakfast and dinner), TV viewing during the meals, and eating family meals in a dining area improvements will be maintained during the follow-up period among families participating in the intervention.

### Study design

The study will be implemented over 12-months as a two-group (intervention; waitlist control) quasi-experimental trial using a staggered cohort design (Table 1). At each of three time periods, separated by 10 weeks, a cohort of 20 families will be recruited. Each cohort will be divided into an intervention and waitlist control group (10 families in each). Consequently, a total of 60 families (30 in the intervention group and 30 in the waitlist control group) will be enrolled. Upon confirmation of study eligibility, a baseline data collection appointment will be scheduled at the participating family’s home or the community center during the two weeks preceding intervention commencement. Data will be collected on the primary food preparing caregiver and all children 4–10 years old. Written caregiver consent and child assent will be obtained. Data will be collected on all outcomes via direct measure and questionnaires at baseline (time point 0, T0), 10-week post-test (time point 1, T1), and 10-week follow-up (time point 2, T2). Repeatability of the intervention (replication) will be evaluated by assessing measures on the waitlist control group at T1 and T2. Assessments will last up to 90 min. A team of trained research staff, blinded from group assignment, will facilitate data collection. Caregiver participants will receive a $25 grocery store gift card at each data collection point for their participation in the research. All study materials and procedures have been approved by the Institutional Review Board at Ohio State University.

**Table 1:**
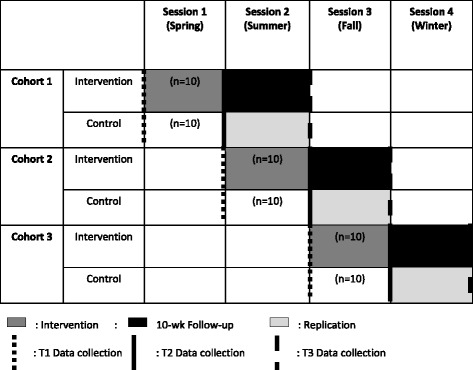
Simple Suppers Intervention Study Design: Two-Group, Staggered Cohort Quasi-Experimental Design

Following baseline data collection, families will decide whether to enroll in either the upcoming 10-week session of Simple Suppers (intervention group) or to wait for 10-weeks (waitlist control group) after which time they would begin the Simple Suppers program. Randomization of families is not feasible because of scheduling conflicts with participating families, the desire of families to participate in the program with families they know, and the need to establish trust with the site/participating families; thus, to preserve sample size and establish trust with the site/participating families, the personal preference of participating families will determine group membership.

### Setting

A faith-based community center will serve as the setting for the Simple Suppers intervention. The question of “who is my neighbor?” is central to the mission and ministries of the center, which has approximately 10,000 visits per month for programming. The most recent service area census tracts demonstrate the following statistics in the center’s immediately surrounding neighborhoods: median household income is $32,307 to $58,490, compared to $51,890 in the broader county; number of families falling below the poverty line ranges from 10.7% to 24.9%, compared to 13.2% in the broader county; higher percentage of racial and ethnic minorities than the county as a whole, with 41.8% being Black compared to 21.2 in the county; and a high percentage of households that are families (58.7%).

### Participants

Participants will be recruited in-person at community center events, center newsletter advertisements, and posters displayed in center. Information on recruitment materials will direct interested families to contact the research team for a screening evaluation to determine study eligibility. To be eligible for inclusion, caregivers should be the primary food preparer in the home; be responsible for at least one child 4–10 years of age; speak English as the primary language in the home; and have lived in the U.S. for at least one year. Families with one or more family members following a restrictive or therapeutic diet will be excluded.

### Intervention

The Intervention Mapping protocol was utilized in the development of the Simple Suppers intervention [16, 17]. Formulation of proximal program objectives occurred as the first step in the mapping process. Based on the current evidence linking family meals with improved child diet and weight status [2–5], the following program objectives were formulated: 1) ‘Increase frequency of family meals prepared in the home (≥5 days/week)’ and 2) ‘Improve child diet quality (significantly increase Healthy Eating Index (HEI) score (*p* < 0.05); increase servings of fruits and vegetables to meet Dietary Guidelines recommendations; significantly decrease daily servings of sugar sweetened beverages (*p* < 0.05)’ (Table 2).

**Table 2:** Overview of formulated program objectives at each level of intervention

Program objective	Level of Intervention	Target group	Performance Objectives
1. Increase frequency of family meals prepared in the home (≥5 days/week)^a^	Individual	Child	PO1. Children participate in cooking activities
	Interpersonal	Caregiver	PO2. Caregivers identify health benefits of regular family meals prepared in the home PO3. Caregivers plan well-balanced weekly dinner menus that include ≥1 svg from 3 of the 5 food groups PO4. Caregivers plan when and where family meals will be served in the home PO5. Caregivers use list for grocery shopping PO6. Caregivers use cost-saving strategies for family meals in the home PO7. Caregivers use time-saving strategies for family meals in the home
2. Improve child diet quality (significantly increase HEI score (*p* < 0.05); increase daily svgs of fruits, vegetables to Dietary Guidelines recommendations;^b^ significantly decrease daily svgs of: SSBs (*p* < 0.05 decrease)^c^	Individual	Child	PO1. Children know health benefits of eating well-balanced meals and snacks PO2. Children participate in planning/preparing well-balanced family meals ≥2x/week
	Interpersonal	Caregiver	PO3. Caregivers know benefits of serving well-balanced meals/snacks PO4. Caregivers serve family meal in the home that include ≥1 svg from 3 of the 5 food groups ≥1x/week PO5. Caregivers serve ≥3 snacks/week that include ≥1 serving from 2 food groups PO6. Caregivers buy food for planned meals/snacks at grocery store

*PO*: Performance objective *HEI*: Healthy Eating Index *SSB*: Sugar sweetened beverage *Svg*: Serving

^a^Measured by asking the question, “During the past 7 days, how many times did all or most, of your family eat dinner together?”[7]

^b^U.S. Departments of Agriculture and Health and Human Services. Dietary Guidelines for Americans, 2010. 7th ed., Washington, DC. December, 2010 [18]

^c^Measured by 24-h dietary recall [29]

Matrices containing the behavioral performance objectives relating to each program objective were created for each level of intervention: individual (child) and interpersonal (caregiver) (Table 2). Development of the performance objectives were guided by the evidence-based 2010 Dietary Guidelines for Americans guidelines for families and children [18]. For example, under program objective 1) (family meals), the performance objective at the individual (child) level was ‘Children participate in cooking activities’ and at the interpersonal (caregiver) level, ‘Caregivers identify health benefits of regular family meals prepared in the home’.

After formulation of performance objectives, a list of personal determinants for each performance objective was generated based on the theoretical foundation of the Simple Suppers program – the Social Cognitive Theory, which posits that behavior change is a function of a reciprocal relationship between personal (e.g., behavioral capabilities and cognitive factors, such as self-efficacy and self-evaluation) and environmental (e.g., norms, modeling, and reinforcement) factors [19, 20]. Next, personal determinants were selected for children at the individual level and caregivers at the interpersonal level based on importance (i.e., strength of the association of the determinant with the behavior) and changeability (i.e., likelihood that the intervention may impact the determinant) [16]. The personal determinants included: behavioral capability; self-efficacy; self-evaluation; and norms, modeling, and reinforcement (Table 3). The performance objectives were then crossed with the selected determinants, which resulted in matrices of change objectives (Tables 3 and 4). The change objectives stated precisely what needs to change in the determinants’ behavioral outcomes in order to accomplish the performance objectives. They were developed using action words and followed by a statement of what is expected to result from the intervention [16, 17]. Because two target groups were selected, two difference matrices of change were developed under each program objective. For example, for program objective 1) (family meals), on the individual (child) level, the performance objective for children that stated ‘Children participate in meal preparation activities’ was crossed with the determinant ‘behavioral capability’, which resulted in the change objective that ‘children practice cooking skills during Simple Suppers and at home’. An example on the interpersonal (caregiver) level, also for program objective 1) (family meals), is as follows: the performance objective for caregivers that stated ‘Caregivers identify health benefits of regular family meals prepared in the home’ was crossed with the determinant ‘behavioral capability’, which resulted in the change objective that ‘Caregivers know benefits of regular family meals prepared at home.’

**Table 3:** Matrix of change objectives by level of intervention for program objective 1 of the simple suppers intervention

Program objective 1: Increase frequency of family meals prepared in the home (≥5 days/week)^a^					
Level of intervention	Performance objectives	Personal determinants			
		Behavioral capability	Self-efficacy	Self-evaluation	Norms, modeling, reinforcement
Individual (child)	PO1. Children participate in meal preparation activities	CO1.1.1 Children practice cooking skills during Simple Suppers and at home CO1.1.2 Children are able to participate in age-appropriate cooking activities at Simple Suppers and at home	CO2.1 Children express confidence in participating in cooking activities	CO3.1 Children are able to determine if they meet their weekly goal for participating in cooking at home	CO4.1.1 Children participate in cooking activities at Simple Suppers family meals 1x/week CO4.1.2 Children increase their participation in cooking at home to ≥1x/week in the home
Interpersonal (caregiver)	PO2. Caregivers identify health benefits of regular family meals prepared in the home	CO1.2.1 Caregivers identify barriers to family meals at home CO1.2.2 Caregivers know benefits of regular family meals prepared at home			
	PO3. Caregivers plan well-balanced weekly dinner menus that include ≥1 svg from 3 of the 5 food groups	CO1.3.1 Caregivers know importance of planning/serving well-balanced dinner menus CO1.3.2 Caregivers know how to plan/serve well-balanced family meals at home	CO2.3 Caregivers express confidence in planning/serving well-balanced family meals	CO3.3 Caregivers are able to determine if they meet their weekly goal for planning/serving well-balanced family meals at home	CO4.3.1 Caregivers learn to plan, prepare and serve well-balanced family meals from Simple Suppers Educators CO4.3.2 Caregivers plan, prepare and serve ≥1 well-balanced family meal at home each week
	PO4. Caregivers plan when and where family meals will be served at home	CO1.4.1 Caregivers know importance of mealtime routines CO1.4.2 Caregivers know strategies to minimize mealtime distractions CO1.4.3 Caregivers plan/establish family mealtime routines	CO2.4.1 Caregiver expresses confidence in establishing mealtime routines at home CO2.4.2 Caregiver expresses confidence in minimizing mealtime distractions	CO3.4.1 Caregivers able to determine if family mealtime routines are being established CO3.4.2 Caregivers able to determine if mealtime distractions are minimized	CO4.4 Caregivers guided by Simple Suppers Educators in establishing family mealtime routines during Simple Suppers group family meals
	PO5. Caregivers use list for grocery shopping	CO1.5.1 Caregivers know benefits of using a grocery list CO1.5.2 Caregivers know how to develop grocery list using planned family meals	CO2.5.1 Caregivers express confidence about developing grocery list CO2.5.2 Caregivers express confidence in using list for grocery shopping	CO3.5 Caregivers able to determine if they meet their goal to develop and use a list for grocery shopping	CO4.5 Caregivers develop weekly grocery list for planned family meals
	PO6. Caregivers use cost-saving strategies for family meals at home	CO1.6 Caregivers know how to use cost-saving strategies to plan/prepare family meals at home	CO2.6 Caregivers express confidence in preparing and serving family meals at home on a budget		
	PO7. Caregivers use time-saving strategies for family meals at home	CO1.7 Caregivers know how to use time-saving strategies to plan/prepare family meals at home	CO2.7 Caregivers express confidence in preparing and servings family meals at home when time is limited		

*PO* performance objective, *CO* change objective, *HEI* healthy eating index, *Svg* serving, *SSB* sugar sweetened beverage

^a^Measured by asking the question, “During the past 7 days, how many times did all or most, of your family eat dinner together?”[7]

**Table 4:** Matrix of Change Objectives by Level of Intervention for Program Objective 2 of the Simple Suppers Intervention

Program objective: Improve child diet quality (significantly increase HEI score (*p* < 0.05); increase daily svgs of fruits, vegetables to Dietary Guidelines recommendations; significantly decrease daily svgs of: SSBs (*p* < 0.05 decrease)^a^					
Level of intervention	Performance objectives	Personal determinants			
		Behavioral capability	Self-efficacy	Self-evaluation	Norms, modeling, reinforcement
Individual (child)	PO1. Children know health benefits of eating well-balanced meals/snacks	CO1.1 Children know health benefits of eating a variety of nutritious foods	CO2.1 Children express confidence in knowing health benefits of eating well-balanced meals/snacks		
	PO2. Children participate in planning/preparing well-balanced family meals/snacks ≥2x/week	CO1.2.1 Children can identify food group sources in meals/snacks CO1.2.2 Children are able to perform age-appropriate coking skills	CO2.2 Children express confidence in participating in meal/snack planning/preparation	CO3.2 Children are able to determine if they meet their weekly goal for participating in family meal/snack preparation	CO4.2.1 Children participate in cooking a well-balanced family meal/snack with peers of the same age 1x/week during Simple Suppers CO4.2.2 Children participate in cooking well-balanced family meals/snacks at home ≥1x/week
Interpersonal (caregiver)	PO3. Caregivers know benefits of serving well-balanced meals/snacks	CO1.3.1 Caregivers identify barriers to offering well-balanced meals/snacks and know strategies to overcome identified barriers CO1.3.2 Caregivers know short- and long-term consequences of not serving well-balanced meals/snacks	CO2.3 Caregivers express confidence in knowing benefits of serving well-balanced meals/snacks		
	PO4. Caregivers serve a family meal that includes ≥1 serving from 3 of the 5 food groups ≥1x/week	CO1.4.1 Caregivers know importance of including a variety of foods in meals CO1.4.2 Caregivers know ≥2 strategies to incorporate foods from 3 food groups into family meals	CO2.4 Caregivers express confidence in planning/preparing well-balanced family meals CO2.4.2 Caregivers express confidence in eating/serving well-balanced family meals	CO3.4.1 Caregivers set goal to serve a family meal that includes ≥1 serving from 3 of the 5 food groups ≥1x/week CO3.4.2 Caregivers monitor goal progress and determine if meeting established goal	CO4.4 Caregivers plan ≥1 family meal/week that includes ≥1 serving from 3 of the 5 food groups
	PO5. Caregivers serve ≥3 snacks/week that include ≥1 serving from 2 food groups	CO1.5.1 Caregivers know importance of eating/serving well-balanced snacks CO1.5.2 Caregivers are able to plan ≥3 snacks/week that include ≥1 serving from 2 food groups	CO2.5.1 Caregivers express confidence in planning well-balanced snacks CO2.5.2 Caregivers express confidence in eating/serving well-balanced snacks	CO3.5.1 Caregivers set goal to serve ≥3 snacks/week that include ≥1 serving from 2 food groups CO3.5.2 Caregivers monitor goal progress and determine if meeting established goal	CO4.5 Caregivers plan ≥3 snacks/week that include ≥1 serving from 2 food groups
	PO6. Caregivers buy food for planned meals/snacks at grocery store	CO1.6.1 Caregivers plan well-balanced family meals and snacks CO1.6.2 Caregivers prepare grocery list using planned meals/snacks	CO2.6.1 Caregivers express confidence in developing grocery list CO2.6.2 Caregivers express confidence in using list for grocery shopping	CO3.6.1 Caregivers set goal to develop and use list for grocery shopping each week CO3.6.2 Caregivers monitor goal progress and determine if meeting established goal	CO4.6 Using list for grocery shopping becomes norm for caregivers

*PO* Performance objective, *CO* Change objective, *HEI* Healthy Eating Index, *Svg* Serving, *SSB* Sugar sweetened beverage

^a^U.S. Departments of Agriculture and Health and Human Services. Dietary Guidelines for Americans, 2010. 7th ed., Washington, DC. December, 2010 [18]

Next, theory-based methods to influence change in the determinants at the individual (child) and interpersonal (caregiver) level were selected based on the theoretical framework of the intervention (Social Cognitive Theory) [19, 21] and in reference to methods described by Bartholomew et al. [16, 17]. For identifying theory-based methods to influence determinants at the interpersonal (caregiver) level, the Adult Learning Theory, which purports that adult learning is most effective when a collaborative, problem-based approach was also referenced [22, 23]. A list of all change objectives that were linked with a specific determinant was made, and the theoretical methods were then matched with the corresponding determinant (Table 5). Finally, practical strategies were designed to put the theoretical methods into practice (Table 5). For example, under the family meals program objective, on the individual (child) level, the result of crossing the performance objective ‘children participate in meal preparation activities’ with the determinant ‘behavioral capability’ was the change objective ‘children are able to participate in age-appropriate cooking skills’. The selected theory-based method that corresponded to the determinant ‘behavioral capability’ in order to achieve the change objective was facilitation. This theory-based method was then translated into a practical strategy. In this case, a practical strategy that was chosen for the method facilitation was to ‘Learn age appropriate cooking skills at each Simple Suppers lesson’. An example on the interpersonal (caregiver) level, also under the family meals program objective, (caregiver) level is as follows: the result of crossing the performance objective ‘Caregivers identify health benefits of regular family meals prepared in the home’ with the determinant behavioral capability was the change objective ‘Caregivers know benefits of regular family meals prepared at home’. The selected theory-based method that corresponded to the determinant behavioral capability in order to achieve the change objective was active learning. This theory-based method was then translated into a practical strategy. In this case, a practical strategy that was chosen for the method active learning was: ‘Educators use the 4A method (participants think about their experience with a topic (Anchor), learn new information (Add), reinforce learning through hands-on activities (Apply), and set goals to utilize new knowledge at home (Away)) to lead weekly caregiver discussions [23, 24].

**Table 5:** Theory-based methods and practical strategies to achieve the change objectives for selected program objectives of the simple suppers intervention

Program objective	Level of intervention	Determinant	Change objective	Theory-based method	Theory	Practical strategy
1. Increase frequency of family meals prepared in the home (≥5 days/week)^a^	Individual (child)	Behavioral capability	CO1.1.1, CO1.1.2	• Facilitation	• SCT	• Learn new age-appropriate cooking skills at each Simple Suppers lesson • Discuss food safety and cleanup with Educators
			CO1.1.1, CO1.1.2	• Vicarious learning	• SCT	• Children divided into three age groups (4–5 years olds; 6–8 years olds; 9–10 year olds) for nutrition education & engagement in food preparation
			CO1.1.1, CO1.1.2	• Mastery experience	• SCT	• Learned food prep skills accrued/practiced over lessons
		Self-efficacy	CO2.1	• Facilitation	• SCT	• Educators provide guidance & feedback as children learn/practice food prep skills
			CO2.1	• Vicarious learning	• SCT	• Participate in cooking activities with peers of the same age
			CO2.1	• Mastery experience	• SCT	• Practice cooking skills learned during Simple Suppers at home
		Self-evaluation	CO3.1	• Self-monitoring	• SCT	• Establish weekly goal during Simple Suppers to practice newly learned cooking skill at home • Weekly goals are reinforced by sharing goal with caregivers during Simple Suppers family meal
			CO3.1	• Feedback	• SCT	• Discuss cooking skills used at home during past week with Educators and peers during Simple Suppers
		Norms, modeling, reinforcement	CO4.1.1, CO4.1.2	• Facilitation	• SCT	• Engage in family meal cooking activities with peers and Educators during Simple Suppers • Decorate/wear aprons for food prep during Simple Suppers and at home • Share cooking skills learned each week with caregivers at start of Simple Suppers group family meals • Lead cleanup at Simple Suppers family meals • Families receive take-home cooking utensil during each Simple Suppers lesson
			CO4.1.1, CO4.1.2	• Mastery experience	• SCT	• Repeated engagement in family meal cooking during Simple Suppers • Weekly goal established to engage in family meal food prep at home
	Interpersonal (caregiver)	Behavioral capability	CO1.2.1, CO1.2.2, CO1.3.1, CO1.3.2, CO1.4.1, CO1.4.2, CO1.4.3, CO1.5.1, CO1.5.2, CO1.6, CO1.7	• Active learning	• ALT	• Educators use 4A method to lead weekly caregiver discussions • Educators engage caregivers in games, meal planning & goal-setting related to weekly lesson topics
			CO1.3.2, CO1.4.2, CO1.4.3, CO1.5.2, CO1.6, CO1.7	• Facilitation	• SCT	• Educators provide resources (e.g., recipe book, coupons, store ads) to plan family meals using skills learned at each lesson
			CO1.2.1, CO1.2.2, CO1.3.2, CO1.4.2, CO1.4.3, CO1.5.2, CO1.6, CO1.7	• Problem solving	• ALT	• Caregivers set weekly goals & discuss successes/challenges with meeting goals with Educators & other caregivers • Educators & caregivers provide suggestions to help peer caregivers overcome challenges preventing them from reaching their goals
			CO1.2.1, CO1.2.2, CO1.3.1, CO1.3.2, CO1.4.1, CO1.4.2, CO1.4.3, CO1.5.1, CO1.5.2, CO1.6, CO1.7	• Vicarious learning	• SCT	• Caregivers acquire new knowledge through peer discussions • Caregivers participate in games, goal-setting & menu planning with peer caregivers
			CO1.3.2, CO1.4.3, CO1.5.2, CO1.6, CO1.7	• Mastery experience	• SCT	• Caregivers plan ≥1 family meal using skills learned each week to practice skills at home
		Self-efficacy	CO2.3, CO2.4.1, CO2.4.2, CO2.5.1, CO2.5.2, CO2.6, CO2.7	• Feedback	• SCT	• Discuss challenges and successes with weekly family meals goal. • Problem solve with peers to overcome challenges
			CO2.3, CO2.4.1, CO2.4.2, CO2.5.1, CO2.5.2, CO2.6, CO2.7	• Social support	• SCT	• Post goal successes and challenges throughout week on Simple Suppers Facebook page. Peers and Educators provide praise/support/encouragement
			CO2.3, CO2.4.1, CO2.4.2	• Modeling	• SCT	• Caregivers plan family meals for upcoming week with peer caregivers during weekly lessons • Caregivers observe Educators facilitating group family meal during weekly lessons
			CO2.3, CO2.4.1, CO2.4.2,	• Mastery experience	• SCT	• Caregivers participate in group family meals during weekly lessons • Caregivers plan and set weekly goals to have family meals at home
		Self-evaluation	CO3.3, CO3.4.1, CO3.4.2, CO3.5	• Self-monitoring	• SCT	• Set individualized weekly SMART goals aligned with lesson topics
			CO3.3, CO3.4.1, CO3.4.2, CO3.5	• Feedback	• SCT	• Goals are reinforced by caregivers sharing their weekly goals • Educators and peers provide feedback/assure appropriateness • Discuss previous week’s goal successes and challenges at beginning of each lesson. Caregivers problem solve together to overcome challenges
		Norms, modeling, reinforcement	CO4.3.1, CO4.3.2, CO4.4, CO4.5	• Facilitation	• SCT	• Simple Suppers group family meals follow routine/establish norm for family meals • Provide weekly take-home cooking utensil to facilitate cooking at home
			CO4.4	• Mastery experience	• SCT	• Educators guide caregivers in establishing mealtime routine during Simple Suppers and at home
Improve child diet quality (significantly increase HEI score (*p* < 0.05); increase daily svgs of fruits, vegetables to Dietary Guidelines recommendations; significantly decrease daily svgs of: SSBs (*p* < 0.05 decrease)^b^	Individual (child)	Behavioral capability	CO1.2.1	• Facilitation	• SCT	• Before Simple Suppers family meals, children name foods from each food group in the upcoming family meal
			CO1.1, CO1.2.2	• Vicarious learning	• SCT	• Discuss food groups and benefits of healthy eating with Educators and peers at Simple Suppers • Learn to cook a variety of foods with peers
			CO1.1, CO1.2.2	• Mastery experience	• SCT	• Children learn food prep skills & become familiar with a variety of food while helping prepare Simple Suppers family meals
		Self-efficacy	CO2.1, CO2.2	• Facilitation	• SCT	• Learn health benefits of foods through interactive discussions & food prep • Engage in planning/preparing well-balanced meals/snacks during Simple Suppers and at home ≥2x/week
			CO2.2	• Vicarious learning	• SCT	• Engage in food prep with peers of the same age • Eat Simple Suppers group family meals with peers
		Self-evaluation	CO3.2	• Self-monitoring	• SCT	• Establish weekly goal during Simple Suppers to try a new food at home • Weekly goal reinforced by sharing goal with caregivers during Simple Suppers family meal
			CO3.2	• Feedback	• SCT	• Discuss new foods tried at home during past week with Educators and peers during weekly Simple Suppers lesson
		Norms, modeling, reinforcement	CO4.2.1, CO4.2.2	• Facilitation	• SCT	• Foods from ≥3 food groups served at Simple Suppers family meals • Eat Simple Suppers family meals with family and peers • Children & caregivers establish weekly goal to engage in preparing well-balanced meals at home ≥1x/week
	Interpersonal (caregiver)	Behavioral capability	CO1.3.1, CO1.3.2, CO1.4.1, CO1.4.2, CO1.5.1, CO1.5.2, CO1.6.1, CO1.6.2	• Active learning	• ALT	• Educators use 4A method to lead caregiver discussions • Caregivers learn skills to serve nutritious meals/snacks through discussions, problem solving, games, meal planning, goal setting
			CO1.4.2, CO1.5.2	• Facilitation	• SCT	• Caregivers plan • Caregivers provided with take-home recipe book of nutritious recipes • Families receive take-home cooking utensil during each lesson
			CO1.3.1, CO1.3.2, CO1.4.1, CO1.4.2, CO1.6.1	• Problem solving	• ALT	• Discuss challenges and successes with serving well-balanced meals/snacks • Problem solve with peers to overcome challenges
			CO1.4.2, CO1.5.2, CO1.6.1	• Vicarious learning	• SCT	• Simple Suppers group family meals contain ≥1 svg from all 5 food groups • Caregivers observe Educators serving/engaging children in preparing well-balanced family meals
			CO1.4.2, CO1.5.2, CO1.6.1, CO1.6.2	• Mastery experience	• SCT	• Caregivers plan ≥1 well-balanced (contains ≥1 svg from 3 food groups) family meal per week during each Simple Suppers lesson using skills acquired each lesson • Learned skills repeated in caregiver family meal planning
		Self-efficacy	CO2.3, CO2.4.1, CO2.4.2,, CO2.5.1, CO2.5.2, CO2.6.1, CO2.6.2,	• Feedback	• SCT	• Discuss challenges and successes with serving well-balanced meals/snacks. • Problem solve as a group to overcome challenges
			CO2.4.1, CO2.4.2, CO2.5.1, CO2.5.2, CO2.6.1	• Social support	• SCT	• Plan weekly family meals with peers during Simple Suppers lessons • Post weekly successes and challenges on Simple Suppers Facebook page. Peers and Educators provide praise/support/encouragement
			CO2.3, CO2.4.1, CO2.4.2	• Modeling	• SCT	• Educators serve Simple Suppers group family meals with ≥1 svg from all 5 food groups • Simple Suppers group family meals eaten with Educators and peers
			CO2.3, CO2.4.1, CO2.4.2	• Mastery experience	• SCT	• Families eat a well-balanced family meal during Simple Suppers group family meals • Caregivers plan ≥1 family meal ≥1 svg from 3 food groups each lesson for upcoming week
		Self-evaluation	CO3.4.1, CO3.4.2, CO3.5.1, CO3.5.2, CO3.6.1, CO3.6.2,	• Self-monitoring	• SCT	• Set individualized weekly SMART goal to serve set number of family meals at home with ≥1 svg from ≥3 food groups • Caregivers plan menus for the number of family meals they made their goal for the upcoming week during Simple Suppers • Goals are reinforced by sharing weekly goal and planned menus during Simple Suppers each week. Educators and peers provide feedback/assure appropriateness
			CO3.4.2, CO3.5.2, CO3.6.2	• Feedback	• SCT	• Discuss previous week’s goal successes and challenges at beginning of each Simple Suppers lesson. Caregivers problem solve together to overcome challenges
		Norms, modeling, reinforcement	CO4.4, CO4.5, CO4.6	• Facilitation	• SCT	• All Simple Suppers group family meals contain ≥1 svg from all 5 food groups • Receive Simple Suppers cookbook with kid-friendly, well-balanced meals

*PO* performance objective, *CO* change objective, *HEI* healthy eating index, *Svg* serving, *SSB* sugar sweetened beverage

*ALT* adult learning theory, *SCT* social cognitive theory

^a^Measured by asking the question, “During the past 7 days, how many times did all or most, of your family eat dinner together?”[7]

^b^U.S. Departments of Agriculture and Health and Human Services. Dietary Guidelines for Americans, 2010. 7th ed., Washington, DC. December, 2010 [18]

The next step was to develop the Simple Suppers curriculum in direct reference to the results produced from the aforementioned Intervention Mapping (Table 6). The initial draft was reviewed by field experts using a nutrition education curriculum assessment tool [25]. Curriculum modifications were then made using reviewer feedback (e.g., incorporating additional hands-on learning activities in the caregiver component to enhance interactive nature of curriculum), after which additional pilot testing occurred and subsequent curricular revisions were made [26].

**Table 6:** Simple Suppers Topics and Goals by Weekly Lesson

Lesson	Topic	Broad goal for upcoming week
1	Making family mealtime fun!	Play 1 family meal-friendly game during mealtime at 2 family meal occasions
2	Planning family meals on a budget	Use 1 cost-saving strategy to plan and serve 1 well-balanced family meal at 1 family meal occasion
3	Timesaving strategies for family meals	Use 1 timesaving strategy to plan and serve 1 well-balanced family meal at 1 family meal occasion
4	Connecting with your child through family meals	Involve child in 1 mealtime activity at 2 family meal occasions
5	Planning well-balanced family meals	Serve a family meal with 1 serving of whole grains, vegetables, and protein at 1 family meal occasion
6	Rethink your drink	Serve 1 well-balanced family meal with low-fat/no sugar added beverages
7	Making healthy cooking tasty & easy	Use 1 healthy cooking method to plan and serve 1 well-balanced family meal at 1 family meal occasion
8	Serving & eating healthy portions	Serve 1 well-balanced family meal with healthy portion sizes at 1 family meal occasion
9	Eating healthy when eating away-from-home	Eat 1 well-balanced, nutritious meal away-from-home at 1 family meal occasion
10	Planning fun & healthy snacks	Serve 2 planned, pre-portioned, well-balanced snacks to your child

Finally, the Simple Suppers program design was developed with feedback from program adopters (faith-based community center staff), implementers, and the target population [27] (e.g., utilizing two (versus one) educators for the caregiver component and incorporating site-based staff into the staffing structure). Each 90-min lesson is delivered weekly over the dinner hour. Session components include: a) interactive group discussion and goal setting with caregivers; b) hands-on activities with children; and c) group family meal with caregivers and children.

### Outcome measures

#### Children and caregivers

##### Diet quality

Dietary intake will be assessed by conducting three, nonconsecutive (two weekdays, one weekend day) 24-h (24 h) dietary recalls using USDA’s 5-step multi-pass dietary recall method [28]. At each data collection time point, the first dietary recall will be conducted during the in-person data collection visit, the remaining two will be conducted via telephone within two weeks of the initial in-person recall. For the child dietary recalls, caregivers will provide assistance, as caregiver-assisted 24 h recalls, collected in this way (i.e., relying on three days and utilizing the multi-pass method), provide the most accurate estimate of dietary intake among children 4 to 11 years of age [29]. Caregiver 24 h dietary recalls will be conducted independently following the child recall(s). Typical daily dietary intake will be determined by averaging dietary intake across the three recalls at each time point to determine daily servings of fruit, vegetables, and SSB. Diet quality will be assessed at each point by calculating a Healthy Eating Index 2010 score using the three 24 h dietary recalls collected [30].

##### Anthropometric assessments

Standardized procedures will be used to assess height and weight on all participating children and caregivers via calibrated stadiometers (Hopkins portable road rod stadiometer) and scales (BFHA-B400SV digital scale), respectively [31, 32]. Body mass index will be calculated using measured heights and weights. Centers for Disease Control and Prevention (CDC) age- and sex-adjusted BMI growth charts will be used to determine BMI z-scores for children to adjust for expected healthy growth and weight gain [31, 33]. Waist circumference will be measured on all participating children and caregivers with a tape measure at the uppermost lateral border of the hip crest (ilium) [31]. To adjust for expected growth among child participants, child WC z-scores will be determined using CDC age- and sex-specific growth charts [34].

##### Blood pressure

Blood pressure will be assessed on all participating children and caregivers via automated, calibrated BP monitors (Panasonic EW3109W). Age-, sex-, and height-adjusted National Heart, Lung, and Blood Institute (NHLBI) charts will be used to appropriately classify child BP [35].

##### Personal determinants

We will also assess immediate intervention targets relating to behavioral capabilities. For child participants, food preparation skills and frequency of involvement will be assessed at each data collection point by caregiver completion of an age-appropriate food preparation skills questionnaire designed to assess both skill ability and frequency of involvement in practicing the skill. Working from an existing validated questionnaire designed to assess child food preparation skills (ability) among 8–10 year olds [7], three versions of the questionnaire (4–5 year old questionnaire; 6–8 year old questionnaire; 9–10 year old questionnaire) were developed to accurately assess child food preparation skills (ability) according to age appropriateness. Assessment of frequency of involvement in practicing each food preparation skill was added to these modified questionnaires. The resulting questionnaires assessing a child’s ability to participate (8 items; 4-point scale; strongly agree to strongly disagree) and frequency of participation (8 items; 5-point scale; 0 times to 7+ times) in age-appropriate food preparation skills (during the past 30 days) included 16 items.

Among caregiver participants, menu planning skills and frequency will be assessed at each data collection point by caregiver completion of an existing menu planning questionnaire [36] to evaluate immediate intervention targets relating to behavioral capabilities. The 9-item menu planning questionnaire, which has demonstrated adequate internal consistency (α = 0.68) and high test-retest reliability (Pearson test-retest = 0.89), asks respondents to rate statements regarding menu planning, meal decision-making, and grocery shopping using a 4-point scale (‘never,’ ‘sometimes,’ ‘often,’ ‘always’).

A key affective variable - caregiver self-efficacy for healthy dietary practices related to family meals - will be assessed using an existing 12-item, 10-point scalar (0 = not at all confident; 10 = extremely confident) questionnaire [37]. The caregiver self-efficacy questionnaire, which will be completed by caregiver participants at each data collection point, has demonstrated high internal consistency (α = 0.88) among a sample of caregivers of 4–6 year old children. Tests of internal consistency will be run on all of the aforementioned questionnaires. Caregivers will also complete a brief food security questionnaire at each data collection point (6-item Short Form of the USDA Home Food Security Survey) [38] and a demographics questionnaire to assess key participant characteristics (age, race/ethnicity, education, employment, income) at baseline.

#### Home environment

##### Family meals

Weekly frequency of shared family dinners, shared family breakfasts, television viewing during family meals, and eating family meals in a dining area will be assessed via caregiver reports with 4, 5-point scalar (0 = never; 5 = 7 times) items from previous family meals research [39, 40].

### Process measures

Feasibility (program dose and fidelity) and acceptability will be assessed prospectively throughout the study as process outcomes. Program dose will be assessed by collecting weekly attendance (family and individual level) and tracking presence of caregiver/child dyads at each weekly lesson. Participants who demonstrate irregular attendance and/or discontinue participation will be contacted to learn underlying reasons for absence. To determine program fidelity, a trained observer will complete a program specific fidelity tool at the end of each weekly lesson, which will include a checklist of key program components, activities, and leader characteristics. Acceptability of the program will be measured with a caregiver-completed 5-item satisfaction survey administered at the end of the 10-week program [41]. At the end of programming, interviews will be conducted with a subset of caregivers to learn their perceptions of program strengths and weaknesses.

### Sample size and data analysis

Sample size was determined by examining the power of the test for comparing increases in frequency of family meals (day per week) of the intervention and waitlist control group. The data used to estimate power come from a previous pilot study, in which the main outcome of interest was the change in frequency of family dinners prepared and eaten at home together (weekly basis) from baseline to post-intervention [42]. Change in frequency of family dinners was used to power the current study because there is strong evidence that it has a downstream effect on the outcome of interest, child BMI [3, 42–44], and there are no previous studies that show a causal effect of family dinners on BMI. Based on these data, assuming 20% attrition [42], with an expected effect size of 0.7071, there will be 80% power to detect a difference in frequency of family dinners of 3 days per week with 30 families per group for a total sample size of 60 families at α = 0.05. Because the sample size in the previous pilot study was small and uncertainty about estimated effect size was large, we used a conservative estimate of effect size (i.e., the lower bound of a 95% confidence interval) for the power calculation.

Data from each of the three cohorts will be pooled and the intervention tested by comparing change (T1-T0) in diet quality, anthropometric measures, and blood pressure of child and caregiver participants in the intervention compared to participants in the waitlist control (hypotheses 1.1 and 2.1). Multiple regression models will be used to determine the association between the difference in the response variables of interest between the intervention and control group, controlling for potential confounders (race/ethnicity, income, cohort, intervention dose), from baseline (T0) to 10-week post-test(T1) and 10-week follow-up (T2). For families in which data will be collected on multiple children, the effect of family will also be controlled by including a random effect for family.

Sustainability of intervention effects will be tested by pooling intervention group data from each of the three cohorts, comparing change (T2-T1) in diet quality, anthropometric measures, and blood pressure among intervention group participants at the end of the 10-week follow-up period (hypothesis 1.2 and 2.2). Intervention replication will be assessed by pooling waitlist control group data from each of the three cohorts, comparing post-program change in diet quality, anthropometric measures, and blood pressure among waitlist control participants (T2-T1) to intervention participants (T1-T0). Significance will be set at *p* < 0.05.

## Discussion

We may encounter challenges engaging and developing trust with the target population, an issue that is common to intervention research with economically disadvantaged families [45–47]. However, this study was designed to minimize this potential barrier by implementing the intervention at a local faith-based community center, which has established relationships with the target population. In addition, this study will engage current staff from the faith-based community centers to serve as educators in delivering the intervention. Grounding the caregiver component in Adult Learning Theory will further enhance our abilities to engage with families, as this approach is designed to present new information in a non-threatening, approachable way.

Another limitation is the lack of randomization study design. Randomization was not appropriate for this study because preserving sample size and developing trust with the site/participating families was paramount [47–49]. We will overcome this limitation by assessing potential between- group differences at baseline and, if identified, will be controlled for in the analyses.
